# Inpatient Neurology Consultations During the Onset of the SARS-CoV-2 New York City Pandemic: A Single Center Case Series

**DOI:** 10.3389/fneur.2020.00805

**Published:** 2020-07-10

**Authors:** Sara Radmard, Samantha E. Epstein, Hannah J. Roeder, Andrew J. Michalak, Steven D. Shapiro, Amelia Boehme, Tommy J. Wilson, Juan C. Duran, Jennifer M. Bain, Joshua Z. Willey, Kiran T. Thakur

**Affiliations:** ^1^Department of Neurology, New York-Presbyterian/Columbia University Irving Medical Center, New York, NY, United States; ^2^Department of Neurology and Epidemiology, Sergievsky Center, Columbia University Irving Medical Center, New York, NY, United States; ^3^Department of Neurology, Division of Child Neurology, New York-Presbyterian/Columbia University Medical Center, New York, NY, United States

**Keywords:** neurology, novel coronavirus, COVID-19, severe acute respiratory syndrome coronavirus 2, inpatient consults

## Abstract

**Objective:** Severe acute respiratory syndrome coronavirus 2 (SARS-CoV-2) primarily causes respiratory illness. However, neurological sequelae from novel coronavirus disease 2019 (COVID-19) can occur. Patients with neurological conditions may be at higher risk of developing worsening of their underlying problem. Here we document our initial experiences as neurologic consultants at a single center quaternary hospital at the epicenter of the COVID-19 pandemic.

**Methods:** This was a retrospective case series of adult patients diagnosed with SARS-CoV-2 who required neurological evaluation in the form of a consultation or primary neurological care from March 13, 2020 to April 1, 2020.

**Results:** Thirty-three patients (ages 17–88 years) with COVID-19 infection who required neurological or admission to a primary neurology team were included in this study. The encountered neurological problems associated with SARS-CoV-2 infection were encephalopathy (12 patients, 36.4%), seizure (9 patients, 27.2%), stroke (5 patients, 15.2%), recrudescence of prior neurological disease symptoms (4 patients, 12.1%), and neuromuscular (3 patients, 9.1%). The majority of patients who required evaluation by neurology had elevated inflammatory markers. Twenty-one (63.6%) patients were discharged from the hospital and 12 (36.4%) died from COVID-19 related complications.

**Conclusion:** This small case series of our initial encounters with COVID-19 infection describes a range of neurological complications which are similar to presentations seen with other critical illnesses. COVID-19 infection did not change the overall management of neurological problems.

## Introduction

Novel coronavirus disease 2019 (COVID-19) is a global pandemic caused by the pathogen severe acute respiratory syndrome coronavirus 2 (SARS-CoV-2) (1). SARS-CoV-2 causes a respiratory illness that primarily manifests in symptomatic individuals as fever, cough, and myalgias. More severe infection leads to pneumonia, acute respiratory distress syndrome (ARDS) or a large systemic inflammatory cytokine response (2), resulting in multi-organ damage.

The full spectrum of neurological manifestations of COVID-19 not fully elucidated. Other coronavirus strains are associated with seizures, encephalitis, and demyelinating syndromes (3). Proposed mechanisms of disease for these strains include entry into the central nervous system through breakdown of the blood brain barrier and invasion through peripheral nerves (3). For SARS-CoV-2 in particular, limited knowledge exists regarding its effect on the nervous system and impact on pre-existing neurological disorders. Case series have primarily reported symptoms of hyposmia, hypogeusia, dizziness, encephalopathy, and delirium (4, 5). Only one case of new-onset seizure has been reported (4). A few studies have noted an increase incidence in stroke with COVID-19 infection (4, 6–8). including in young adults (9).

With the rapid spread of SARS-CoV-2, neurologists will increasingly be consulted to guide care for patients with existing neurological conditions and new neurological symptoms due to COVID-19. We conducted a single-center retrospective review of our first experiences with COVID-19 infection in inpatients with or without pre-existing neurological disorders. We document 33 patients who had neurological symptoms while hospitalized with COVID-19 infection and summarize the associated laboratory and ancillary data performed. We discuss unique cases that raise questions about the nuances of management in a neurological population.

## Methods

### Patients

Adult inpatients diagnosed with COVID-19 infection by nasopharyngeal swab reverse transcription polymerase chain reaction (RT-PCR) and required neurological evaluation by consultation or admission for primary neurological care were included in this single-center retrospective case-series study at Columbia University Irving Medical Center (CUIMC)-New York Presbyterian Hospital. Cases from March 13, 2020 to April 1, 2020. Clinical information was gathered by patient chart review, including demographics, medical history, clinical presentation, clinical course, radiographic imaging, laboratory values, hospital duration, therapeutic agents, and outcome. Pulmonary disease includes pre-existing obstructive lung disease, restrictive lung disease, and/or chronic tobacco use. Cardiovascular disease includes hyperlipidemia or hypercholesterolemia, hypertension, coronary artery disease (CAD), peripheral arterial disease, diabetes mellitus, and morbid obesity. Renal disease is defined as pre-existing chronic kidney disease or end-stage renal disease requiring dialysis. Immune suppression is defined as autoimmune disorder on immunosuppressive therapy, immunodeficiency from HIV/AIDS, or history of cancer on chemotherapy. Encephalopathy is defined as depressed consciousness, fluctuations in consciousness, or impaired consciousness leading to language comprehension deficits excluding aphasia.

### Standard Protocol Approvals and Patient Consents

This study was approved by the Institutional Review Board (IRB) of CUIMC. Informed consent was obtained for the two cases delineated in detail. Informed consent was not required by our institutional IRB for the remainder of the participants.

### Statistical Analysis

Statistical associations were calculated using mean, median, range, and standard deviation. All statistical analyses were carried out using SAS (version 9.4, trademarks of SAS Institute Inc., Cary, NC, USA).

## Results

### Patients

Thirty-three patients were included in this study of which 1 was near adult age (17 years). The mean age of patients was 56.1 (±17.1) years (Table 1). Twenty-two patients (66.7%) had a known neurological condition, including epilepsy (*n* = 6), ischemic stroke (*n* = 5), dementia (*n* = 5), developmental delay (*n* = 4), myasthenia gravis (*n* = 2), and multiple sclerosis or other central autoimmune disease (*n* = 2). Seventeen (51.5%) had cardiovascular comorbidities, 5 (15.2%) pulmonary disease, and 7 (21.2%) renal disease (Table 1). Ten (30.3%) were immunocompromised due to transplant (*n* = 2), cancer with or without chemotherapy (*n* = 7), and autoimmune disorder on immunosuppression (*n* = 1) (Table 1). These cases represented 3.9% of inpatients who had tested positive for SARS-CoV-2 during the 2-week time course.

**Table 1:** Patient demographics, medical history, neurological clinical presentation, COVID-19 laboratory markers, therapeutic interventions, and outcomes.

**Patient demographics & clinical presentation**	
**Age (y), mean (SD), median, range**	56.1 (17.1), 60, 17–88
**Sex**	
Female	13 (39.4%)
Male	20 (60.6%)
**Ethnicity**, ***n*** **(%)**	
White	10 (30.3%)
Hispanic	14 (42.4%)
African American	6 (18.2%)
Unknown	3 (9.1%)
**Neurological symptom**, ***n*** **(%)**	
Encephalopathy	12 (36.4%)
Seizure	9 (27.2%)
Stroke	5 (15.2%)
Recrudescence	4 (12.1%)
Neuromuscular	3 (9.1%)
**Medical History**, ***n*** **(%)**	
Pulmonary disease	5 (15.2%)
Cardiovascular disease	17 (51.5%)
Renal disease	7 (21.2%)
Immune Suppression	10 (30.3%)
**Intubation required**	8 (24.2%)
**Laboratory values, mean (SD)**	
Peak ferritin	1438.8 (1685.0)
Peak IL-6	82.2 (111.9)
Peak D-dimer	6.2 (6.9)
Peak procalcitonin	15.7 (63.6)
Peak C-RP	146.7 (99.8)
**Therapeutic interventions, mean (%)**	
Hydroxychloroqine	15 (45.5%)
Azithromycin	11 (33.3%)
Remdesivir	2 (5.3%)
Tocilizumab	2 (6.1%)
**Outcomes, mean (SD)**	
Length of hospital stay (d)	16.6 (16.8)
Death	12 (36.4%)
Discharged from hospital	21 (63.6%)
Average age at death (y)	61.4 (10.6)

*y, year; d, day; SD, standard deviation; n, number; WBC, white blood count; IL-6, interleukin-6; C-RP, C-reactive protein*.

### Clinical Presentation

The most frequent reason for neurology consultation was encephalopathy (*n* = 12, 36.4%). Encephalopathy was attributed to toxic/metabolic derangements, systemic infection, or concern for cytokine storm. Continuous EEG (cEEG) was obtained for 7 (21.2%) patients to rule out seizure as cause of the encephalopathy. Continuous EEG monitoring showed triphasic waveforms in one patient with hepatic encephalopathy (case 18) and one patient with acute renal failure due to septic shock (case 16).

**Figure 1:**
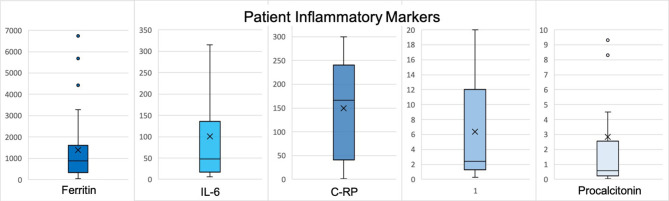
Reported values represent peak levels detected during hospitalization. X represents the mean and the line represents the median. Normal data values based on CUIMC laboratory: WBC 3.12–8.44 x 10(3)/μL, % Lymphocytes 18.5–50.5, ferritin 30–400 ng/mL, IL-6 <5 pg/mL, procalcitonin <0.08 μg/mL FEU, C-RP <3.0 mg/L, D-dimer <0.80 ug/mL FEU. H, Hydroxychloroquine; Az, Azithromycin; R, Remdesevir; T, Tocilizumab; *, given as outpatient; **, patient with known tracheostomy and required ventilator support.

The second most frequent reason for neurology consultation was seizure (*n* = 9, 27.2%), of which 6 (66.7%) patients had first seizure of life (Table 2). Three patients with first seizure of life had cEEG monitoring which showed frontotemporal seizures, parasagittal seizures, and marked attenuation (cases 12, 13, and 21, respectively). Only one patient with first seizure of life had a history of neurological disease, which was possible anoxic brain injury from ventricular fibrillation arrest (case 21). Five (83.3%) patients with first seizure of life had inflammatory marker data. All had elevated C-RP >100 milligram per liter (mg/L), four (80%) ferritin >1,000 nanogram per milliliter (ng/mL), and four (80%) IL-6 >30 picogram per mL (pg/mL) (Figure 1). Two patients with focal seizures had no evidence of structural lesion on CT head. MRI brain with and without contrast was normal for the pediatric first seizure of life patient (case 1). All patients with first seizure of life were treated with levetiracetam except for the pediatric patient who was discharged home on no antiepileptic medications.

**Table 2:** Neurological and non-neurological symptom onset and clinical presentation.

**No**.	**Age(y)/sex**	**Symptom duration, d**	**COVID-19 symptoms**	**Pertinent medical history**	**Neurological history**	**Neurological problem from COVID-19**
1	17/F	2	F, C	–	–	Seizure
2	25/F		F, C, H, S, V	Obesity, IC	Myasthenia gravis	Generalized weakness
3	28/M	3	F, C, M	CKD; IC	Steroid myopathy, possible myasthenia	Dysarthria
4	31/M		C	–	Autism, epilepsy, intellectual disability	Seizure
5	33/M	4	F, C. HA	–	–	Acute Headache (olfactory groove meningioma)
6	39/M	7	F, C, M	Ca, IC	–	Seizure
7	40/M	4	F, C	–	Lennox-gastaut syndrome	Encephalopathy, seizures
8	45/M	3	C	CAD, sickle trait	–	Left thalamic stroke
9	50/M	2	F, C, S	Asthma	–	Encephalopathy, left leg weakness
10	50/F	7	F, C, S	HTN, ESRD	–	Encephalopathy, Seizure
11	51/F	2	F, C, S	Obesity; IC	RRMS	Recrudescence
12	51/M	1	S, CP	Ca, sickle trait, IC	–	Seizure
13	56/M	2	F, C, H, S, AMS	ILD, lung transplant, IC	–	Seizure
14	57/M	6	F, C, HA	Ca, IC	Face numbness and loss of taste	Worsening symptoms/recrudescence
15	57/M		CP	HTN, DM2, CAD	–	Multifocal strokes and systemic thromboses s/p tPA and thrombectomy
16	60/M		F, S	HTN, DM2, CAD, ESRD on iHD	Stroke	Encephalopathy
17	60/F	5	M	HTN, ESRD on iHD	SAH, epilepsy	Encephalopathy
18	61/M		C, AMS	Ca, IC	–	Encephalopathy
19	62/M		F, C, V	HTN, DM2	–	Encephalopathy
20	64/F	2	C, S	DM2, CAD, CKD	Stroke	Encephalopathy
21	65/M	12	F, S, V, D	CAD, prior VF arrest	Possible anoxic brain injury	Seizure
22	67/M		C, D	HTN	HA, Unruptured aneurysm, prior stroke	TIA
23	68/M	2	AMS	DM2, COPD, HTN	TBI, epilepsy	Seizure
24	69/M	Onset after admission	F 1 week into hospitalization	Smoker, Lymphoma of hard palate, IC	Extension of tumor into skull	Encephalopathy, worsening face weakness
28	70/M	3	F, C, M	DM2, HTN, HLD	Prior lacunar ischemic strokes	Recrudescence
25	70/M	1	V, AMS	Abdominal surgery	Childhood meningitis, epilepsy	Seizure
26	70/F	Onset after admission	F 3 days into hospitalization	–	Dementia	Right ICH/IVH/cortical SAH and left hemiparesis
27	72/F	3	F, C, S	OSA, COPD, lung Ca, single lung transplant, IC, CKD	–	Encephalopathy
28	72/M	1	F, M	–	PD	Worsening rigidity and encephalopathy
29	74/F	Onset after admission	AMS	Ca (remission)	Antibody negative autoimmune encephalitis	Encephalopathy
30	78/F	5	F, S, AMS	HTN, CKD	Dementia	Subdural hematoma
31	81/F	1	F, C, M, AMS	CAD, DM2, HTN	Stroke, dementia, meningioma	Encephalopathy
32	88/F	3	C, AMS	CAD, HTN, DM2, CKD	Stroke, dementia, epilepsy	Encephalopathy

*y, years; d, days; F, fever; C, cough; T, tachypnea; H, hypoxia; S, shortness of breath; V, vomiting; M, malaise; HA, headache; CP, chest pain; AMS, altered mental status; ID, intellectual disability; D, diarrhea; OSA, obstructive sleep apnea; COPD, chronic obstructive pulmonary disease; Ca, cancer; HTN, hypertension; DM, diabetes mellitus; ILD, interstitial lung disease; ESRD, end-stage renal disease; CKD, chronic kidney disease; iHD, intermittent hemodialysis; VF, ventricular fibrillation; RRMS, relapsing-remitting multiple sclerosis; ICH, intracerebral hemorrhage; IVH, intraventricular hemorrhage; SAH, subarachnoid hemorrhage; TIA, transient ischemic attack; IC, immunocompromised; TBI, traumatic brain injury; PD, Parkinson disease; tPA, tissue plasminogen activator; s/p, status post*.

Five (15.2%) patients presented with acute cerebrovascular insults due to ischemia (*n* = 3; ages 45, 57, and 67 years), intracerebral hemorrhage (ICH)/intraventricular hemorrhage (IVH)/subarachnoid hemorrhage (SAH) (*n* = 1), and subdural hematoma (SDH) (*n* = 1) (Table 2). All patients with ischemic strokes had cardiovascular risk factors (cases 8, 15, and 22). One patient had sickle cell trait (case 8).

Recrudescence of a prior neurological condition occurred in 4 (12.1%) patients. Recrudescence was diagnosed if a patient had a prior neurological deficit that worsened in the setting of infection but improved back to baseline after symptomatic treatment of underlying infection.

### Laboratory and Radiographic Data

**Figure 2:**
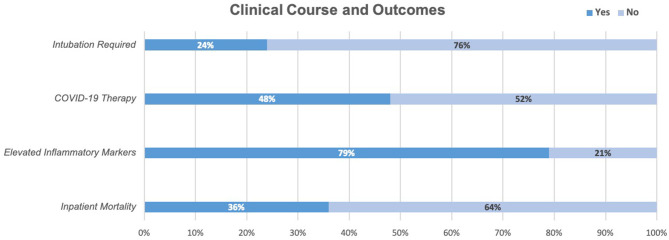
In our cohort, 24% of patients required intubation, 48% received at least one COVID-19 therapy (45% trialed on Hydroxychloroquine, 33% Azithromycin, 6% Tocilizumab, 5.3% Remdesevir), 79% had two or more elevated inflammatory markers above the upper limit of normal (C-RP, IL6, Ferritin). At the time of chart review, 36% had died while in the hospital and 64% had been discharged.

Of the inflammatory marker levels obtained, ferritin was elevated in 75% (*n* = 21/28) of patients, IL-6 in 57% (*n* = 12/21), d-dimer in 65% (*n* = 15/23), and C-RP in 90% (*n* = 26/29) (Figure 2).

Brain imaging was obtained for 20 (60%) patients, including 3 (7.6%) patients who underwent MRI. Relevant findings included acute SDH, ICH/IVH/SAH, acute ischemic strokes (*n* = 2), and meningioma with vasogenic edema (*n* = 1). The remainder showed chronic changes or were unremarkable. Of note, one patient who had several months of hyposmia and facial numbness presented with worsening of the symptoms. MRI brain with and without contrast was unremarkable with no abnormal contrast enhancement (case 14).

In an attempt to reduce exposure and preserve personal protective equipment (PPE), CSF testing was only pursued if there was another indication. As a result, CSF was only obtained in one case highlighted below for autoimmune encephalitis.

### Patient Outcomes

Twenty-one (63.6%) patients were discharged from the hospital and 12 (36.4%) died. One 70-year-old male known epilepsy and intellectual disability from childhood meningitis presented with breakthrough seizures and died due to presumed cytokine storm and multiorgan failure. One patient with relapsing-remitting multiple sclerosis on natalizumab who presented with worsening bilateral leg weakness died due to severe ARDS and shock.

### Case 29

A 74-year-old woman with a history of remote breast and pancreatic cancers, recent varicella zoster virus (VZV) radiculitis, and possible antibody negative autoimmune encephalitis was transferred to our institution for worsening mental status manifested as stupor and global aphasia. CSF studies were notable for a white blood cell count 12 per microliter (μL) (99% lymphocytes), red blood cell count 32/μL, glucose 59 mg per decaliter (mg/dL), protein 38 mg/dL, negative meningitis/encephalitis panel (BioFire Diagnostics, Salt Lake City, UT) that includes VZV, and negative paraneoplastic panel (Mayo Clinic Laboratories). MRI brain with and without contrast demonstrated stable periventricular and subcortical white matter T2 hyperintensities. Continuous EEG was negative for seizures. She was treated with high dose IV steroids for 3 days and IV immunoglobulin (IVIG) for 5 days resulting in improvement of her mental status. However, 5 days after completing IVIG, she developed an increasing oxygen requirement, lethargy, and worsening aphasia. Chest X-ray revealed multifocal pneumonia, and nasopharyngeal swab was positive for SARS-CoV-2 on hospital day 17. Initial laboratory work-up was notable for new lymphopenia and elevated inflammatory markers. Her mental status decline was attributed to COVID-19 infection and recrudescence of her autoimmune encephalitis, so repeat LP was deferred. Azithromycin and hydroxychloroquine were started for SARS-Cov-2-directed experimental therapy. One day after initiation, she had significant improvement in her mental status and oxygenation. Nine days after symptom-onset, she returned to her neurological baseline.

### Case 15

A 57-year-old man with history of hypertension, type 2 diabetes mellitus, and CAD was admitted with chest pain and found to have a myocardial infarction for which he underwent a 3-vessel coronary artery bypass grafting (CABG). On post-operative day one (hospital day 6), a stroke code was called for acute onset right-sided weakness and obtundation. Following CT head, he was given tissue plasminogen activator (tPA). CT angiogram demonstrated vertebrobasilar occlusion. He underwent successful thrombectomy of the basilar artery. Angiogram at time of the thrombectomy demonstrated interval development of a new right internal carotid artery occlusion, which was also successfully treated. Follow-up CT head revealed a complete left posterior cerebral artery stroke with other smaller posterior circulation strokes with petechial hemorrhage in the right basal ganglia. Within 12 h of tPA administration, he developed thrombosis of the right brachial artery. He remained intubated. On hospital day 7, CT chest revealed bilateral consolidation concerning for pneumonia. He developed ARDS and tested positive for SARS-CoV-2 on hospital day 11. He sustained multi-organ failure due to mixed cardiogenic and septic shock and died on hospital day 25.

## Discussion

The most common reasons for neurological evaluation in the setting of COVID-19 infection at our institution were encephalopathy, seizure, and stroke. Most of the patients in our case series developed neurological symptoms several days after COVID-19 symptom-onset and demonstrated elevated inflammatory markers. It is shown that patients with moderate to severe disease requiring hospitalization are more likely to develop a secondary, delayed hyperinflammatory state with elevated cytokine levels (2, 10). However, we are not able to comment on how these levels compare to COVID-19 patients without neurologic symptoms in our inpatient population, COVID-19 patients in the outpatient setting, nor to healthy controls. This will be important to evaluate in future studies.

Severe SARS-CoV-2 infection can lead to impaired consciousness (4). Many factors can attribute to toxic-metabolic encephalopathy in patient with COVID-19, including hypoxia, organ dysfunction, and metabolic abnormalities (11). Many patients who require intubation and critical care also require prolonged sedation, immobilization, hospitalization, and social isolation, which also may contribute to toxic-metabolic encephalopathy (11). A postmortem brain examination has demonstrated brain edema and neuronal degeneration (12); however, these findings can be seen with hypoxia and metabolic abnormalities (13). Direct invasion of SARS-CoV-2 has not been demonstrated. The ability of our study to sample CSF in the initial stages of the pandemic at our institution were limited.

Acute symptomatic seizures can occur in the setting of systemic illness and sepsis (14), proposing a systemic inflammatory response leading to a decreased seizure threshold. The six patients who presented with first-time seizure of life had high inflammatory markers known to be elevated in COVID-19 infection, which suggests that these seizures could be categorized as provoked seizures in the setting of robust systemic inflammatory response. Other coronavirus infections have been associated with seizures (15). CSF was not obtained in our cases, so we could not exclude direct neuroinvasion or encephalitis as the cause of seizures.

Infection is a known trigger for stroke, potentially contributing up to one-third of ischemic strokes (16). Respiratory infections in particular have been associated with acute cerebrovascular events, most notably with influenza-like viral illnesses (17, 18). Stroke is emerging as a potential complication of infection with SARS-CoV-2. The incidence and relative risk of stroke from COVID-19 infection remains unknown. Retrospective studies in different countries have reported an incidence of 2.5–5.7% for acute stroke (4, 6, 7). Case series in the United States have also demonstrated acute stroke occurring in young adults (9) and in patients with only mild respiratory symptoms from COVID-19 (8). These studies did not have comparative controls.

The mechanisms whereby COVID-19 may increase stroke risk include the following: Hypercoagulability (as demonstrated in part by elevated D-dimer) may contribute. Systemic inflammation and a cascade of pro-inflammatory cytokines (as demonstrated in part by elevated IL-6) may be a mediator between infection and hypercoagulability and may also destabilize atheromatous plaque (4, 19, 20). Coronavirus-related cardiac injury may lead to embolic strokes (21). Direct viral invasion of the vascular wall via binding at and depletion of the angiotensin-converting enzyme 2 (ACE2) receptor may generate endothelial dysfunction, vasoconstriction, and stroke (22).

As in prior literature reviewing neurological manifestations of COVID-19 infection, our case series included instances of both ischemic stroke and intracranial hemorrhage, although our patients also had other cardiovascular risk factors for stroke. Due to our limited sample, we cannot comment on SARS-CoV-2 increasing the risk of acute stroke.

This study had several limitations. First, it was a retrospective study including patients at a single urban academic medical center. Only 33 cases were studied. While the goal of this study was to provide an account of our early experiences with the neurological manifestations of COVID-19 infection, these factors limit the generalizability of our data. This study included only cases for which neurology was consulted or involved in primary care of the patients, so these cases do not represent all neurological manifestations of COVID-19 infection. These have been reported elsewhere (4). Although in some cases diagnostic tests may have better elucidated mechanisms of neurologic symptoms in our patients, these were not uniformly performed due to either patient illness severity, PPE scarcity, or healthcare exposure. This was particularly true for lumbar punctures given the risk of prolonged exposure during the procedure. We therefore were unable to rule out alternative neurological infections as a cause of seizure, including in the three immunocompromised patients. Lastly, since this is a short-term study period, long-term outcomes are not known.

Here we present the most common neurological problems during the first two weeks of the SARS-CoV-2 outbreak at our institution. COVID-19 infection did not change the overall management of neurological problems. All deaths were directly related to severe sepsis, ARDS, or cytokine storm causing refractory shock. Future studies will investigate if SARS-CoV-2 has direct neurological toxicity requiring alternative management strategies.

## Data Availability Statement

The raw data supporting the conclusions of this article will be made available by the authors, without undue reservation.

## Ethics Statement

The studies involving human participants were reviewed and approved by Columbia University Irving Medical Center Institutional Review Board. Written informed consent to participate in this study was provided by the participants' legal guardian/next of kin. Written informed consent was obtained from the individual(s) for the publication of any potentially identifiable images or data included in this article.

## Author Contributions

SR, SE, HR, AM, and SS have full access to all of the data and takes full responsibility for the data presented, accuracy of the data analysis and interpretation, the conduct of the research design, and drafting of the manuscript for intellectual content. TW and AB has full access to all of the data and contributed to the conduct of the research design and statistical analysis. JD has full access to all of the data and takes full responsibility for the data presented and accuracy of the data analysis and interpretation. JB has full access to all of the data and takes full responsibility for the data presented, accuracy of the data analysis, interpretation, and revising the manuscript for intellectual content. JW and KT were responsible for conduct of the research design, analysis and interpretation of the data, and revising the manuscript for intellectual content. All authors contributed to the article and approved the submitted version.

## Conflict of Interest

The authors declare that the research was conducted in the absence of any commercial or financial relationships that could be construed as a potential conflict of interest.
